# Tietze’s Syndrome

**DOI:** 10.31138/mjr.33.4.467

**Published:** 2022-12-31

**Authors:** Jozélio Freire de Carvalho

**Affiliations:** Institute for Health Sciences, University of Bahia, Salvador, Bahia, Brazil

**Keywords:** Tietze’s syndrome, costochondritis, spondyloarthritis

**Figure 1.**
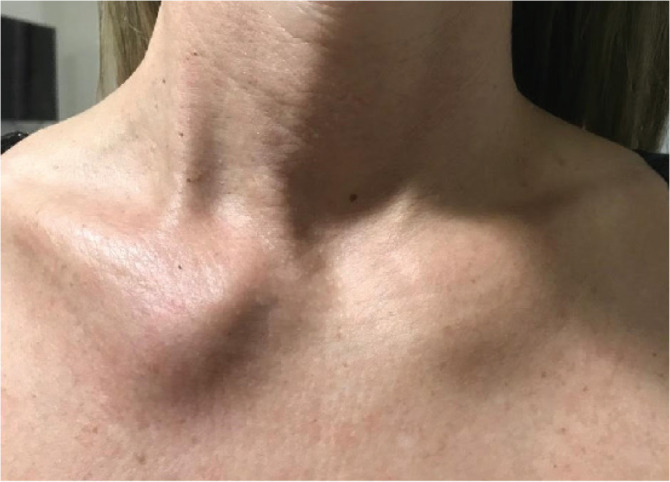
Erythematous and nodular augmentation of the right sternoclavicular joint.

**Figure 2.**
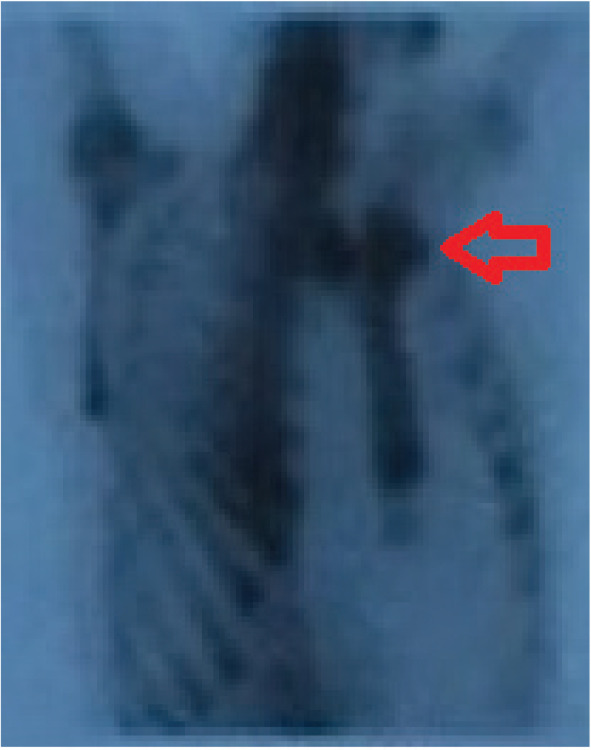
Bone scintigraphy showing hyper-uptake of the sternoclavicular joints

A 52-year-old woman with a past medical history of dyslipidaemia, using ezetimibe 10mg plus simvastatin 20mg/day. She started pain and erythema on her right sternoclavicular joint (SCJ) in September 2018. She denied any other symptoms. She went to an orthopaedist, and she received betamethasone depot twice, with pain improvement. Her physical examination demonstrated arthritis (erythema, warmth, and oedema) of both SCJ more prominent to the right side (**Figure 1**). Sacroiliac manoeuvres and skin were normal. Bone scintigraphy showed a marked hyper-uptake of the sternoclavicular joints (**Figure 2**), no sacroiliitis. The X-ray showed augmentation of soft tissues of the SCJ. Computed tomography showed some subchondral cyst o the SCJ, no evidence of a tumour. Laboratory test revealed C-reactive protein of 0.32mg/dL (normal range [nr]: <1mg/dL), erythrocyte sedimentation rate of 8mm/1^st^ hour (nr: < 12mm/1st hour) and 25-OH-vitamin D of 20.4 ng/mL (nr: > 30ng/mL). Antinuclear antibodies, rheumatoid factor, and HLA-B27 were absent. Blood cell count, blood chemistry, total and fraction cholesterol, triglycerides, parathyroid hormone, calcium, and phosphorus were normal. A diagnosis of Tietze’s syndrome (TS) was determined, and a prescription with non-hydrolysed collagen type II 40mg/day plus Move (Boswellia serrata-20% AKBA) was initiated (she was allergic to several non-steroidal anti-inflammatory drugs). After 4 months, she was oligosymptomatic, and Move was excluded.

TS is a benign and self-limiting joint disease, commonly the sternocostal, sternoclavicular, or costochondral joints. The TS diagnosis is based on physical examination (an increase of palpation tenderness in the affected joint), laboratory tests (an increase of inflammatory parameters), and imaging studies. Its pathophysiology is unknown but seems to involve microtrauma.^1^ Differential diagnosis of Tietze’s syndrome is based on excluding costal cartilage inflammation, coronary syndrome, and inflammatory changes in the lung and pleura. Most commonly, the treatment is conservative with analgesics and non-steroidal anti-inflammatory drugs. In resistant cases, corticosteroid infiltration is a possibility.^2^ Additional therapy options include avoiding movements that cause pain and intercostal blockade, local lidocaine infiltration, and, rarely, surgical resection of the affected joint.
